# Natural occurrence of aflatoxin residues in fresh and sun-dried meat in Nigeria

**Published:** 2010-11-19

**Authors:** Gbonjubola Oyero Olufunmilayo, Akeeb Bola Oyefolu

**Affiliations:** 1 Department of Biology, The Polytechnic, Ibadan, Nigeria,; 2 Department of Microbiology, Lagos State University, Lagos, Nigeria^2^

**Keywords:** Aflatoxin, Carcinogenic, Meat, Mycoflora, Mycotic diseases, Mycotoxigenic, Thin layer chromatography, Nigeria

## Abstract

**Introduction:**

In recent times, food safety and security have generally remained basic human needs, therefore because of the largely unregulated nature of the Nigerian markets, coupled with the poor housing and feeding conditions to which animals are subjected in the abattoirs, a survey for assessing potential mycotoxin exposure through meat consumption was undertaken.

**Methods:**

Eighty Samples of meat bought randomly from 5 major markets distributed in 5 local government areas of Oyo state , Nigeria were analysed for contaminating mycoflora using the plate count and micromorphological methods, while aflatoxin detection and quantification was by Thin Layer Chromatography (TLC).

**Results:**

Mycological analysis of samples revealed a higher contamination level in the sun-dried samples. Eighteen fungi species belonging to 8 genera, namely, *Aspergillus, Penicilliu, Alternaria, Cladosporium, Fusarium, Neurospora, Rhizopus and yeast* were identified. The predominant genus *Aspergillus* yielded 7 species while the potential toxicogenic fungi represented 38% of the isolated mycoflora. The genera requiring higher water activity for growth ( *Alternaria, Fusarium* and *yeast*) were not obtained from the dried meat. Aflatoxins B1, B2, G1 and G2 were detected in all the samples analysed. The fresh samples with the exception of the total aflatoxin G (AFG) in kidney gave the highest mean concentrations for all aflatoxins, also an exceptionally high aflatoxin content was found in all the kidney samples.

**Conclusion:**

Aflatoxin detection in meat should be addressed urgently to avert the possible adverse health effects like aflatoxicosis, exacerbated malnutrition, suppression of growth and immune functions on consumers. Also the animal health inspectors should pay more attention to the feeding conditions of the animals on farm and the abattoirs.

## Introduction

Meat refers to meat flesh, skeletal muscles, connective tissue or fat and others than meat flesh, including brain, heart, kidney, liver, pancreas, spleen, thymus, tongue and tripe that is used as food, excluding the bone and bone marrow and it contains high biological value protein and important micronutrients that are needed for good health throughout life [1]. However in Nigeria, meat consumption is correlated with the affluence of individuals, although man’s dependence on plant materials when consumed directly could support a substantially greater population than the animal products derived when fed to animals; this is true with respect to the total available calories, protein and other nutrients needed to sustain life.

Raw materials for animal feeds are exposed to microbial contamination at the time of their cultivation, processing, storage and distribution, while chemical composition of food, properties of the outside environmental and specific growth requirements determine the type of microorganisms and the course of physico-chemical reaction in the contaminated food [2].

In general, mycotoxin exposure is a critical problem in the hot and humid low income countries where poor methods of food handling and storage are common. In spite of occasional high profile incidents of acute poisoning outbreak, mycotoxins have not been widely prioritized from a public health perspective [3-4]. However attention has only been paid, largely to meet the stringent import regulations on mycotoxin contamination in the richer nations of the world. Humans become exposed directly by consuming contaminated commodities or indirectly by consuming products from animals that have ingested mycotoxin contaminated feeds [5]. While animal slaughter slabs, insufficient cleaning and disinfection of working areas, instruments and other equipment contribute greatly to meat contamination by toxigenic moulds, the conditions of the environment in the stores, refrigerators and shops are very suitable for the development of moulds inside the products, but more frequently on the surface of various sorts of meat and meat products [2,6]. Feeds and foods are often contaminated with various moulds and when the temperature and relative humidity are optimal after contamination, there is also a risk of mycotoxin production [7]. Relatively low water activity (aw < 0.9) and low pH-values (pH < 6.0) are particularly favorable for mould development [8].

Although mycotoxin contamination of food commodities is a global problem, the developed world through the application of modern agricultural practices and legislatively regulated food processing and marketing system have greatly reduced exposure in these populations [9]. The contrary is the case in developing countries where populations live largely on commodities from local markets where regulations relating to mycotoxin control and consumer protection rarely exist; even when they are available they are not strictly enforced. As a result, mycotoxins presence in food represents a constant health risk for man.

Meat as a source of animal protein is consumed heavily in Nigeria and is also is recommended by nutritionists as a major source of protein for growing children, the convalescent, the expectant mothers and the aged. Therefore, the objective of this was to determine the various contaminating fungi on meat, assaying for mycotoxin residues and ascertaining the general safety of meat consumed in Northern and Eastern parts of Nigeria; in order to create awareness on the presence, concentrations and distributions of mycotoxins in meat. It could also form the scientific basis for promulgation of regulations important in the decision-making process to establish meaningful limits for mycotoxins in foods, most especially meat and meat products [9].

## Method

**Sample preparation, isolation, enumeration and identification of mycoflora**

Five major markets for bulk purchase and retail of foodstuffs and animal produce brought largely from the Northern and Eastern parts of Nigeria were selected for this study. The markets (aleshinloye, bodija, moniya, oja-oba and oje) distributed in 5 local government areas (Onireke, Ibadannorth, Akinyele, Ibadan- northwest, and Ibadan- northeast respectively) are known for wide patronage by inhabitants of the South-western states. Ten samples each of fresh and sun-dried pieces of cow beef and edible organs (liver, kidney and heart) were purchased randomly from these markets. They were placed in labeled sterile polythene bags and then transferred immediately to the laboratory for mycological and mycotoxin residue analysis. Ten grams of sample was diluted with 90mL of physiological saline and homogenization was achieved using mortar and pestle. Two hundred microlitres (200Ll) of ten-fold serially diluted homogenized sample was inoculated onto potato dextrose agar (PDA) incorporated with antibiotics. Incubation was at 25 degrees Celsius for 3-5 days. Fungal enumeration was by plate count while mycoflora identification was by micromorphological characteristics [10].

**Extraction, detection and quantification of aflatoxins in meat samples**

A thin layer chromatographic (TLC) method previously described by Oyero and Oyefolu, 2009 [9] was used and done in triplicates. Aflatoxin content of 5g of meat was extracted with 2.5ml of distilled water and 25ml of chloroform while shaking on a mechanical shaker. Ten milliliter (10ml) of the filtrate obtained was evaporated to dryness on water bath. A mixture of the reconstituted extract (0.2ml) and chloroform (1ml) spotted on pre-coated TLC plate was developed in chloroform/acetone (9:1) tank and detection was done alongside standards under the UV light at 360nm. For quantification, 0.8ml of extract was applied to a 0.5 Lm preparative TLC plate as a band. Following plate development and UV examination, areas containing the toxin was scrapped and extracted in chloroform. The optical density of 3ml of the extract was then determined on a UV spectrometer at 360nm. Aflatoxin concentration was then calculated using the absorbance values for the sample and standard.

## Results

The mycoflora content of the samples ranged from 6.5 x 105 to 2.2 x 106 CFU and 1.25 x 106 to 2.95 x 106 CFU in the fresh and sun-dried samples respectively. On the overall, the contaminating mycoflora load was found to be higher in the sun-dried samples. Mycological analysis of the meat samples resulted in the isolation of 18 fungal species, belonging to 8 genera, namely, *Aspergillus, Alternaria, Fusarium, Cladosporium, Penicillium, Neurospora, Rhizopus and Yeast*. Three of these ( *Aspergillus, Fusarium* and *Penicillium*) are potential mycotoxin producers (Table 1). Apergillus, the predominant genus was identified into 7 species, these included *A. niger, A. tamari, A. fumigatus, A. terreus, A. flavus, A. citrinum, and A. sydowii*. The genus *Penicillium* and *Rhizopus* yielded 2 species each i.e. *P. Oxalicum, P. chrysogenum, R. nigrificans and R. oligosporus*. Others however gave only one specie each. However it was observed that no Penicillium and Neurospora Species were isolated from the fresh sample while members of the genera *Alternaria, Fusarium and Yeast* were also not recovered from the sun-dried meat.

The potential health hazard that may be associated with the consumption of the various meat parts was assessed by the analysis for mycotoxin residues. The two types of aflatoxins (AFB, AFG) commonly found in animal tissues were found in varying concentrations. The fresh samples, with the exception of the total AFG (AFG1, AFG2) in kidney gave the highest mean concentrations for all the aflatoxins detected (Table 2). This ranged from 0.0217 to 0.0852Lg/kg and 0.0267 to 0.0741 Lg/kg for total AFB (AFB1 and AFB2) and AFG respectively. This was followed by the beef, liver, and heart with 0.0559, 0.0339, 0.0217 Lg/kg for AFB and 0.0741, 0.0414, and 0.0267 Lg/kg for AFG respectively. However the total aflatoxin contents of dried samples were lower, ranging from 0.0029 to 0.0758Lg/kg and 0.0031 to 0.1413 Lg/kg for AFB and AFG respectively. In addition an exceptionally high aflatoxin concentration was found in all the kidney samples.

## Discussion

The determination of the level of fungal contaminants in the samples analysed revealed a high microbial load in the sun- dried meat. This may not be unconnected with the fact that during the sun drying process, meats are spread openly in the sun and on bare concrete floors, this often allow contamination by dust. However, the mycoflora on the fresh meat may have been derived from contaminants in water, air, floors and surroundings of the slaughter house, as well as the instruments used during the slaughtering and processing into table meat for sale. Also the situation of the meat processing plants, the slaughter of animals, insufficient cleaning and disinfestations of working areas, instruments and other equipment contributes greatly to meat contamination by toxigenic moulds [12].

The mycoflora of the meat were identified into the *Aspergillus , Alternaria, Fusarium, Cladosporium, Rhizopus, Penicillium, Neurospora* and *Yeast* genera. The incidence of the various genera has been documented by several workers [2,13-17]. The mycotoxin producing ability of many of the species have also been demonstrated in vitro [6,17-19]. Consequently the presence of moulds in meat and meat products could cause a decrease in their biological value due to the enzymatic degradation of meat components [2]. However their metabolic interactions with bacterial pathogens has played important role in the outbreak of food-borne illnesses [20]. The potential health hazards of the mycoflora need not be overemphasized. This is because the fungi following inhalation/ingestion are capable of surviving in the respiratory and intestinal tracts, where they may continue to grow and produce their toxins. These have been implicated in the pathogenesis of several mycotic and aflatoxin induced diseases in man. The *Alternaria, Aspergillus, Cladosporium* and *Penicillium* genera have been implicated as a cause of rhinitis and severe asthma [21] while the contributory role of *Fusarium* and *Penicillium* species in systemic respiratory disease as well as opportunistic infections involving various body sites by the Aspegillus and Rhizopus sp have been reported. The *Aspergillus sp* especially is particularly dangerous to the asthmatics and those with weakened immune systems [22-24]. The non-isolation of the *Fusarium, Alternaria* and *Yeast* species from the dried meat samples however may be due to the fact that they require a moderately high moisture content of 22-23 % [25]. This requirement however was only found or met in the fresh samples.

Aflatoxins commonly associated with animal tissue (AFB1, AFB2, AFG1, and AFG2) were found in all the samples analysed. In nature, several mycotoxins co-occur in contaminated commodity [26] and so the occurrence of AFB and AFG was not surprising. On the overall, total aflatoxin content was higher in the fresh samples. This may have arisen from the fact that the animals were fed on aflatoxin contaminated food till the point of slaughter. It has been suggested that a withdrawal of animals from contaminated feeds onto mycotoxin free diets for 3-4 weeks could have allowed a sufficient withholding period to clear the muscles and organs from toxins [27]. Although the concentrations of the aflatoxins determined were insignificant when compared to the African maximum permissible limits of 5mg/kg and 20mg/kg for AFB1 and total aflatoxins in foods respectively [28]; the overall impact of mycotoxins on health however is dependent on the concentrations and duration of exposure, the toxicity of the compound, the body weight of the individual, the synergistic effects of mycotoxin, environmental factors and other effects [29-30].

The AFB1 and AFG1 detected in samples are proven human carcinogens and are thus classified as Group 1 carcinogens with AFB2 as Group 2B probable human carcinogens [31]. Documented evidence have shown that AFB1 exposure exacerbates protein calorie malnutrition, thereby suppressing growth as well as immunoglobulin (IgA) response to some vaccine challenges, among the African children [32-33]. Consequently the resultant effects of AFB1 exposure may include growth and immune function suppression.

## Conclusion

This study confirmed that meat generally sold in Nigeria are grossly contaminated by various fungi species including the potentially mycotoxigenic ones. The determination of aflatoxins though in acceptable concentrations should urgently be addressed with a view of ensuring the safety of the consumers.

## Competing interests

The authors declare no competing interests.

## Authors’ contributions

**AOBO** was involved in study design, laboratory analysis of samples and wrote the initial draft of the manuscript; **OGO** lufunmilayo was involved in study conception, design, sample collection, laboratory analysis and writing of the final manuscript.

## Figures and Tables

**Table 1:** Distribution of mycoflora of 80 meat samples collected from 5 major markets distributed in 5 local government areas of Oyo State, Nigeria

**Fungal genera / Species**								
	*Aspergillus*	*Alternaria*	*Fusarium*	*Cladosporium*	*Rhizopus*	*Penicillium*	*Neurospora*	*Yeast*
	+	+	+	+	+	-	-	+
	*niger*	*Alt. spp*		*Clad. spp*	*nigrificans*			
**Fresh meat**	*tamarii*							
	*fumigatus*							
*terreus*								
	*+*	*-*	*-*	*+*	*+*	*+*	*+*	*-*
	*niger*	*-*	*-*	*Clad. sp*	*nigrificans*	*Oxalicum*	*Neurospora spp*	
	*flavus*				*oligosporus*	*Chrysogenum*		
**Dried meat**	*terreus*							
	*citrinium*							
	*tamarii*							
	*sydowii*							

**Table 2:** Aflatoxin content of 80 meat samples collected from 5 major markets distributed Nigeria

	**Mean aflatoxin (AF) concentration (8g/kg)**						
		**Mean aflatoxin (AF) concentration (8g/kg)**	**N**	**Median viral load baseline (A) x 104copies (95%CI)**	**Median Viral load after Malaria (B)x104copies (95% CI)**	**Log difference in median viral load log(B-A)**	P*
		**B1**	**B2**	**Total AFB**	**G1**	**G2**	**Total AFG**	
	Liver	0.0714	0.0165	0.0339	0.0200	0.0214	0.0414
**Fresh meat**	Kidney	0.0435	0.0417	0.0852	0.0360	0.0347	0.0707
	Beef	0.0100	0.0117	0.0217	0.0134	0.0133	0.027
	Heart	0.0285	0.0274	0.0559	0.0367	0.0374	0.0741
	Liver	0.0021	0.0010	0.0031	0.0014	0.0017	0.0031
**Dried meat**	Kidney	0.0348	0.0410	0.0758	0.0670	0.0743	0.1413
	Beef	0.0013	0.0016	0.0029	0.0027	0.0017	0.0044
	Heart	0.0143	0.0136	0.0279	0.0240	0.0310	0.0550

B: Aflatoxin B, G: Aflatoxin G
